# Effects of temperature and incubation time on the *in vitro* expression of proteases, phospholipases, lipases and DNases by different species of *Trichosporon*

**DOI:** 10.1186/2193-1801-3-377

**Published:** 2014-07-26

**Authors:** Henri Donnarumma Levy Bentubo, Olga Fischman Gompertz

**Affiliations:** Institute of Health Sciences, Universidade Cruzeiro do Sul, Rua Doutor Ussiel Cirilo, 225, Zip Code: 08060-070, São Paulo, SP Brazil; Department of Microbiology, Immunology and Parasitology, Federal University of São Paulo, Rua Botucatu, 862, Edifício das Ciências Biomédicas, 8th floor, Zip Code: 04060-064, São Paulo, SP Brazil

**Keywords:** Proteases, Phospholipases, Lipases, DNases, *Trichosporon* spp, Virulence factors

## Abstract

Fungi produce a broad spectrum of enzymes capable of degrading different substrates in nature. When the substrate is the tissue of a vertebrate host, these enzymes acts as a fungal virulence factor that increases the pathogenicity of the fungus. *Trichosporon* yeasts are emerging pathogens that infect immunocompromised patients. Little is known about the virulence characteristics of these fungi. The aim of this research was to characterize the behavior of protease, phospholipase, lipase and DNase production in different species of *Trichosporon*, with a focus on the influence of incubation temperature on the expression of these enzymes. Classical methodologies were used in all of the experiments, and the results were statistically analyzed. The proportions of the samples that produced each type of enzyme were as follows: lipases (95.5%), phospholipases (56.8%), proteases (50,0%) and DNases (38.6%). The incubation temperature was an important factor in the expression of enzymatic activity, and it influenced the incubation period of each species. Although these data concerning the enzymatic activity expressed by isolates of *Trichosporon* are valuable, further research is warranted to completely characterize this new pathogen, as well as *in vivo* studies to determine the roles of these enzymes in the pathogenesis of trichosporonosis.

## Background

Virulence factors allow pathogenic agents to grow and establish themselves in host tissues. Dimorphism, thermotolerance, the expression of cell wall components, the presence of a capsule and the secretion of enzymes are all major effects of virulence factors identified in fungi (Naglik et al. 2004; Jain et al. 2008; Colombo et al. 2011). Fungi express a broad spectrum of enzymes capable of degrading various components of host tissues. The study of extracellular enzymatic activity has contributed to the understanding of some of the ecological characteristics of pathogenic yeasts and molds (Gácser et al. 2007). Extracellular enzyme activity is well studied in *Candida* yeasts, but little is known about members of the phylum Basidiomycota, such as the genus *Trichosporon* (Coutinho and Paula 2000; Naglik et al. 2004; Ichikawa et al. 2004). Although *Trichosporon* spp. represent the third most common yeast responsible for invasive fungal infections, few published studies have characterized the virulence of this fungus (Pagano et al. 2006; Chagas-Neto et al. 2008).

The genus *Trichosporon* is associated with superficial infections, such as white piedra or onychomycosis, which can serve as reservoirs for invasive infection. Trichosporonosis is the name assigned to the invasive form of the diseases caused by *Trichosporon* spp.. Malignant hematological diseases, such as leukemias and lymphomas, and organ transplantation, especially bone marrow transplantation, cause patients to become more prone to trichosporonosis due to the profound immune depression associated with those conditions (Chagas-Neto et al. 2008). Most cases of *Trichosporon* infection are reported in medical centers in North America (33.9%), Europe (27.6%) and Asia (23.3%) (Menezes et al. 2012). Mortality rates can reach 80%, even after the initiation of antifungal therapy (Nucci et al. 2010; Colombo et al. 2011).

The disseminated form of trichosporonosis is characterized by a nonspecific fever that does not yield to conventional antibiotic therapy. The infection progresses rapidly, culminating in multiple organ failure. The lungs and kidneys become overwhelmed. Patients with pulmonary involvement have dyspnea and cough, sometimes with blood. Renal failure manifests as hematuria and proteinuria (Heslop et al. 2011). The involvement of the skin is characterized by erythematous papules on the trunk and extremities, which may develop blisters with necrotic centers (Walsh et al. 1993; Hsiao et al. 1994). Histopathological findings from experimental animal infections and cases of human infection have shown that the lesions of trichosporonosis are due to embolisms produced by the vascular invasion of the fungus. Therefore, multifocal areas of infarction are also observed in the affected organs (Nahass et al. 1993; Chagas-Neto et al. 2008).

Currently, 50 *Trichosporon* species are recognized, 16 of which are considered etiological agents of infection: *Trichosporon asahii*, *T. asteroides*, *T. coremiiforme*, *T. cutaneum*, *T. chiarelli*, *T. dermatis*, *T. dohaense*, *T. domesticum*, *T. faecale*, *T. inkin*, *T. japonicum*, *T. jirovecii*, *T. lactis*, *T. montevideense*, *T. mucoides* and *T. ovoides* (Pagnocca et al. 2010; Colombo et al. 2011). *Trichosporon* species isolated from clinical samples are associated with specific types of infection. *T. inkin* and *T. ovoid*es are common agents of white piedra of the pubic hair and scalp, respectively, whereas *T. asteroides* and *T. cutaneum* are associated with lesions of the skin and nails (Chagas-Neto et al. 2008). *T. asahii* is the species most frequently associated with invasive infections, followed by *T. mucoides* and *T. inkin* (Fleming et al. 2002; Madariaga et al. 2003). The objective of this research was to investigate the influence of temperature on the kinetics of the expression of proteases, phospholipases, lipases and DNases by different species of *Trichosporon* and to establish laboratory parameters for this emerging opportunistic yeast.

## Results

**Figure 1:**
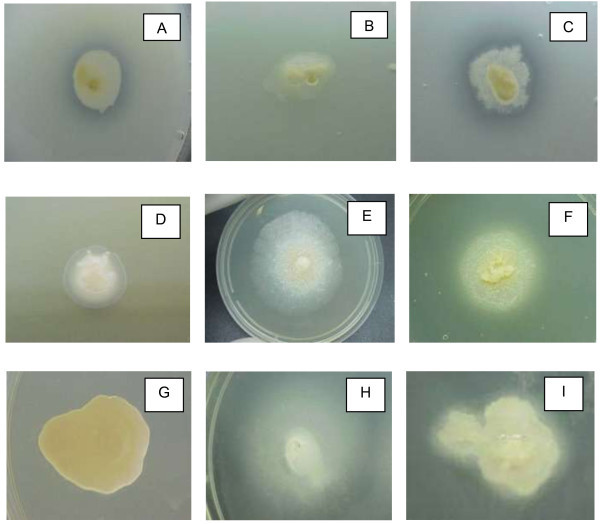
**Expression of extracellular enzyme activity by 44 samples** ***Trichosporon*** **spp.** In the first line: activity of protease in *C. albicans* (ICB 12A) **(A)** *T. asahii* (ICB 439/98) not producing **(B)** and a positive strain of *T. inkin* (CBS 5585) **(C)**. The second line shows a positive sample for production of phospholipase **(D)** and negative samples **(E)** and positive **(F)** to lipase, respectively. In G, note the absence of halo of degradation by DNase **(G)** and two other producers samples DNAse in **H** and **I**.

**Figure 2:**
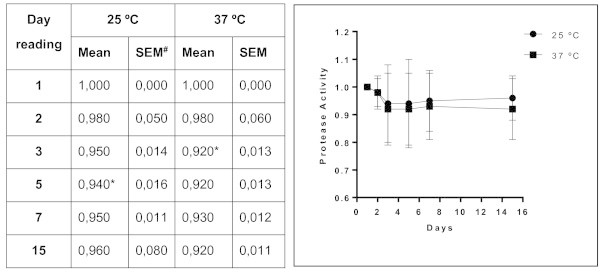
**Mean values and standard error of protease activity in isolates of** ***Trichosporon*** **spp.** Profile of expression of protease by *Trichosporon* spp. over 15 days of incubation; each point on the graph corresponds to the enzyme production rate observed in the 1st, 2nd, 3rd, 5th, 7th and 15th days of incubation under two different temperatures, respectively. *Index "optimum" temperature for enzyme production (*P* < 0.0001 vs Time 1) over the time. # SEM = standard error of the mean.

**Figure 3:**
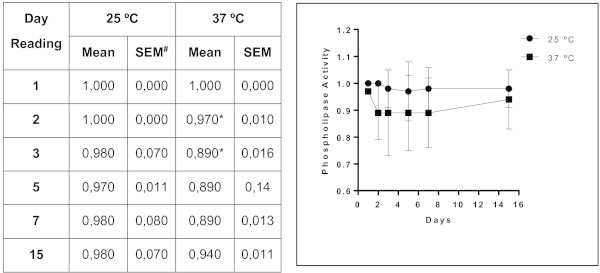
**Mean values and standard error of phospholipase activity in isolates of** ***Trichosporon*** **spp.** Profile of the expression of phospholipase by *Trichosporon* spp. over 15 days of observation; each point on the graph corresponds to the enzyme production rate observed in the 1st, 2nd, 3rd, 5th, 7th and 15th days of incubation under two different temperatures, respectively. *Index "optimum" temperature for enzyme production (p <0.0012 vs. Time 1) over the time. # SEM = standard deviation of the mean.

**Figure 4:**
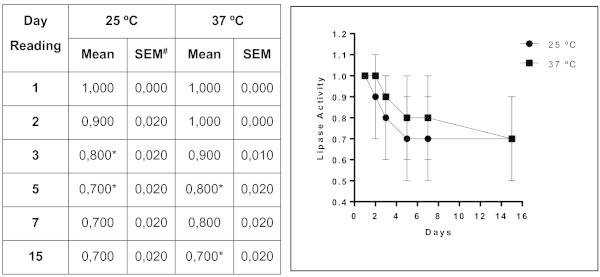
**Mean values and standard error of lipase activity in isolates of** ***Trichosporon*** **spp.** Profile of the expression of lipases by *Trichosporon* spp. over 15 days of observation; each point on the graph corresponds to the enzyme production rate observed in the 1st, 2nd, 3rd, 5th, 7th and 15th days of incubation under two different temperatures, respectively. *Index "optimum" temperature for the enzyme production (p <0.0001 vs. Time 1 and 2) over the time. # SEM = standard error of the mean.

**Figure 5:**
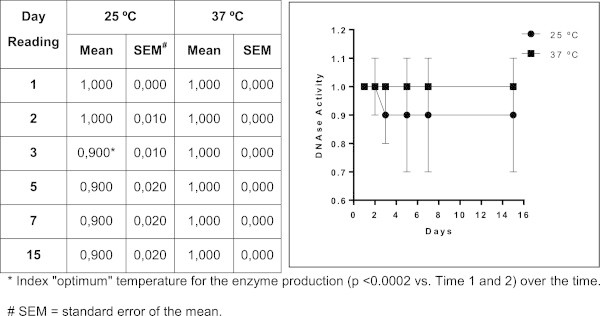
**Mean values and standard error of the activity of DNase in isolates of** ***Trichosporon*** **spp.** Profile of DNases production by *Trichosporon* spp. over 15 days of observation; each point on the graph corresponds to the enzyme production rate observed in the 1st, 2nd, 3rd, 5th, 7th and 15th days of incubation under two different temperatures, respectively. *Index "optimum" temperature for the enzyme production (p <0.0002 vs. Time 1 and 2) over the time. # SEM = standard error of the mean.

Of the forty-four isolates of *Trichosporon* spp. analyzed, 22 (50%) exhibited protease activity. This activity was strongly positive in approximately 41% of these 22 samples. Phospholipase activity was observed in 56.8% of the samples, with 25% of them categorized as strongly positive. Almost all (95.5%) the isolates of *Trichosporon* spp. exhibited lipase activity, and 71% of them were strongly positive. DNase activity was detected in 17 isolates, and more than half (58.8%) were strongly positive. The enzyme activity of samples of *Trichosporon* spp. is presented in Figure 1 and in Table 1. A significant difference between enzyme expression levels at room temperature and at 37°C was observed for proteases (*p <* 0.0001), phospholipases (*p <* 0.0012), lipases (*p <* 0.0001) and DNases (*p <* 0.0002). The higher temperature favored the expression of proteases and phospholipases. The activity of these enzymes was detected in representative numbers of isolates after three and five days of incubation, respectively. Incubation at room temperature (25°C) produced a noticeable delay in the expression of proteases. Furthermore, there was a significant difference between the temperatures in the expression of phospholipases on the second day of incubation (*p <* 0.0012). Room temperature was found to be ideal for the expression of lipase and DNase . When the days of incubation were compared, we observed differences in the amount of lipase (*p* < 0.0001) and DNAse (*p* < 0.0002) expression on the third day of incubation compared with the previous days. The kinetics of enzyme expression in the 44 *Trichosporon* isolates are presented in Figures 2, 3, 4, 5.

**Table 1:** **General profile of 44 samples of** ***Trichosporon*** **spp. production of extracellular enzymes**

Sample profile	Proteinase		Fosfolipase		Lipase		DNAse	
	N	(%)	N	(%)	N	(%)	N	(%)
**(-)**	22	(50,0)	19	(43,2)	2	(4,5)	27	(61,4)
**(+)**	22	(50,0)	25	(56,8)	42	(95,5)	17	(38,6)
**(++)**	9	(40,9)	6	(24,0)	30	(71,4)	10	(58,8)

**(-)** = Negative samples, **(+)** = samples that expressed enzyme activity and **(+ +)** = samples considered strongly positive. Were considered the best temperature and time of reading for enzyme expression.

**Figure 6:**
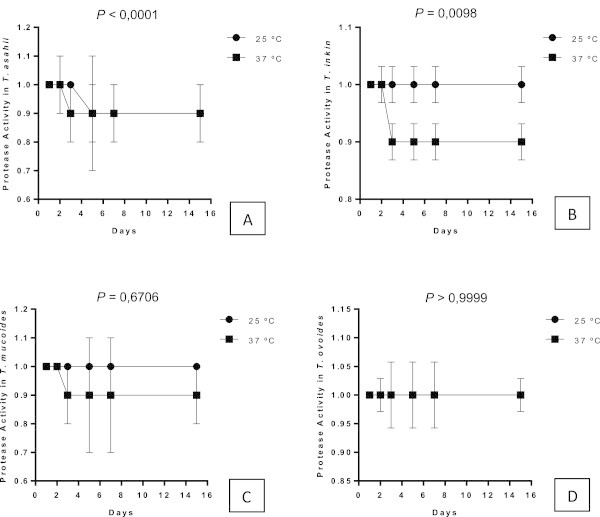
**Protease expression by different species of the yeasts of the genus** ***Trichosporon*** **over 15 days of incubation at different temperatures; each point on the graph corresponds to the average rate of enzyme production seen in the 1st, 2nd, 3rd, 5th, 7th and 15th days of observation, respectively.** The profiles of *T. asahii* **(A)** *T. inkin* **(B)** *T. mucoid*es **(C)** and *T ovoides* **(D)**. The *P* value represents a statistically significant difference between the two incubation temperatures over time.

**Figure 7:**
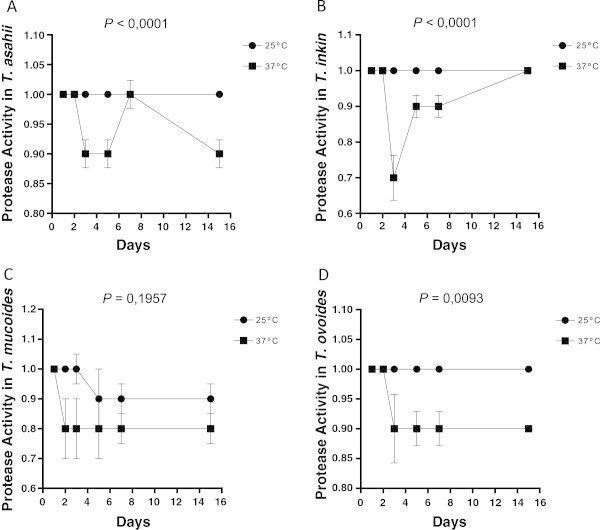
**Phospholipase expression by different species of the yeasts of the genus** ***Trichosporon*** **over 15 days of incubation at different temperatures; each point on the graph corresponds to the average rate of enzyme production seen in the 1st, 2nd, 3rd, 5th, 7th and 15th days of observation, respectively.** The profiles of *T. asahii* **(A)** *T. inkin* **(B)** *T. mucoid*es **(C)** and *T. ovoides* **(D)**. The *P* value is a statistically significant difference between the two incubation temperatures over time.

**Figure 8:**
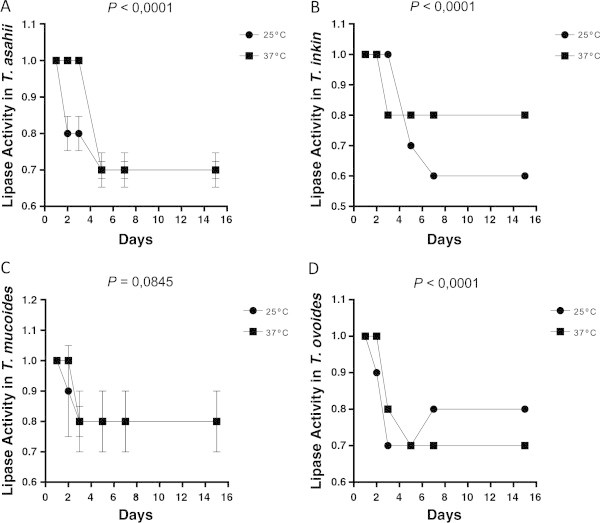
**Lipase expression by different species of the yeasts of the genus** ***Trichosporon*** **over 15 days of incubation at different temperatures; each point on the graph corresponds to the average rate of enzyme production seen in the 1st, 2nd, 3rd, 5th, 7th and 15th days of observation, respectively.** The profiles of *T. asahii* **(A)** *T. inkin* **(B)** *T. mucoid*es **(C)** and *T. ovoides* **(D)**. The *P* value is a statistically significant difference between the two incubation temperatures over time.

**Figure 9:**
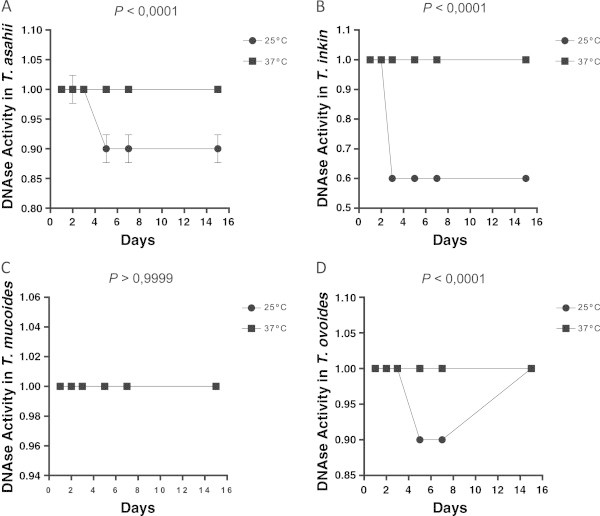
**DNase expression by different species of the yeasts of the genus** ***Trichosporon*** **over 15 days of incubation at different temperatures; each point on the graph corresponds to the average rate of enzyme production seen in the 1st, 2nd, 3rd, 5th, 7th and 15th days of observation, respectively.** The profiles of *T. asahii* **(A)** *T. inkin* **(B)** *T. mucoid*es **(C)** and *T. ovoides* **(D)**. The *P* value is a statistically significant difference between the two incubation temperatures over time.

When the species were analyzed separately, protease activity was detected in 66.7% (12/18) of *T. asahii*, 50% (5/10) of *T. inkin* and 50% (2/4) *T. mucoides* and 25% (3/12) of *T. ovoides* isolates. Strongly positive expression was evident in six of the *T. asahii* samples (33.3%), two of *T. ovoides* (16.7%) and one of *T. inkin* (10%) (Table 2). Although protease activity in *T. asahii* has been detected both at 25°C and 37°C, there was a significant difference between these temperatures (*p <* 0.0001). A large number of samples incubated at 37°C expressed protease activity on the third day, whereas activity was not detected until after five days when the samples were incubated at room temperature. *Trichosporon inkin* did not present enzyme activity when incubated under room temperature, preferring 37°C (*p* = 0.0098). The incubation temperature did not affect the expression of this enzyme by isolates of *T. mucoid*es (*p* = 0.6706) or T. *ovoides* (*p* > 0.9999), as shown in Figures 6, 7, 8, 9.

**Table 2:** **Expression of extracellular enzymes, in absolute numbers, expressed by different species:** ***T. asahii*** **(18),** ***T. inkin*** **(10),** ***T. mucoid*** **es (4) and** ***T. ovoides*** **(12), regardless of the incubation temperature**

Species	Proteinase			Fosfolipase			Lipase			DNAse		
	(-)	(+)	(++)	(-)	(+)	(++)	(-)	(+)	(++)	(-)	(+)	(++)
*T. asahii*	6	12	6	12	6	3	0	18	10	10	8	2
*T. inkin*	5	5	1	4	6	1	1	9	7	3	7	7
*T. mucoides*	2	2	0	1	3	1	1	3	2	4	0	0
*T. ovoides*	9	3	2	2	10	1	0	12	11	10	2	1
**Total**	**22**	**22**	**9**	**19**	**25**	**6**	**2**	**42**	**30**	**27**	**17**	**10**

**(-)** = Negative samples, **(+)** = samples that expressed enzyme activity and **(+ +)** = samples considered strongly positive. Were considered the best temperature and time of reading for enzyme expression.

Phospholipase activity was positive in 33.3% (6/18) of the *T. asahii*, 60% (6/10) of the *T. Inkin,* 75% (3/4) of the *T. mucoid*es and 83.3% (10/12) of the *T. ovoides* isolates. Strongly positive enzyme activity was detected in 16.7% (3/18) of the *T. asahii*, 10% (1/10) of the *T. inkin*, 8.3% (1/12) of the *T. ovoides* and 25% (1/4) of the *T. mucoides* isolates (Table 2). Incubation at 37°C significantly influenced the expression of phospholipases in *T. asahii* (*p <* 0.0001), *T. inkin* (*p* < 0.0001) and *T. ovoides* (*p* = 0.0093). There was no significant difference between the two incubation temperatures for the expression of phospholipases in *T. mucoid*es (*p* = 0.1957). A significant number of *T. asahii* (6/18), *T. inkin* (6/10) and T*. ovoides* isolates (9/12) expressed enzymatic activity on the fifth day of incubation, whereas activity was detected at three days in the isolates of *T. mucoid*es. A statistical analysis revealed a significant difference in the expression of phospholipases after three days of incubation among *T. asahii*, *T. inkin*, and *T. ovoides* (Figures 6, 7, 8, 9).

Forty-two (95.45%) samples of *Trichosporon* spp. exhibited lipase activity. All the isolates of *T. asahii* (18/18) and *T. ovoides* (12/12) showed positive lipase activity. *T. inkin* and *T. mucoides* were positive in 90% (9/10) and 75% (3/4) of the samples, respectively (Table 2). The following percentages of the samples of the examined species were strongly positive: *T. asahii*, 55.5% (10/18); *T. inkin*, 70% (7/10); *T. mucoides*, 50% (2/4); and *T. ovoides*, 91.7% (11/12). Significant numbers of samples of *T. asahii* (18/18) and *T. ovoides* (12/12) expressed lipase activity on the fifth day of incubation at room temperature. The samples of *T. inkin* and *T. mucoides* expressed this activity earlier, after three days of incubation, although incubation temperature produced no statistically significant effect on the expression of lipases in *T. mucoides* (p = 0,0845) (Figures 6, 7, 8, 9).

*T. asahii* was positive for DNase activity in 44.4% (8/18) of the isolates, *T. inkin* [70% (7/10)] and *T. ovoides* [16.7% (2/12)] (Table 2). The same isolates showed strongly positive enzyme activity in 11.1% (2/18) of *T. asahii*, 70% (7/10) of *T. inkin* and 8.3% (1/12) of *T. ovoides* isolates. None of the isolates of *T. mucoides* showed DNase activity. A substantial number of the *T. asahii* samples (8/18) exhibited DNase activity after 7 days of incubation. In the isolates of *T. inkin* (7/10) and *T. ovoides* (2/12), DNase activity was observed after 5 days of incubation. In all cases, room temperature was found to be the most favorable temperature for the expression of DNase activity, except for *T. mucoides* isolates that did not express enzyme activity in either temperature (Figures 6, 7, 8, 9).

## Discussion

Different populations of microorganisms that constitute the microbiota of humans, other animals and the environment adopt dynamic interactive mechanisms in response to competition to survive and evolve. In this context, the modulation of virulence mechanisms is essential to the development of opportunistic microorganisms (Tosta 2001). The close relationship between a parasite and its host sometimes drives adaptations in both organisms to promote their successful coexistence; in some cases, this process leads to the attenuation of virulence (Forattini 2001).

Virulence can be expressed through numerous mechanisms with the common goal of promoting the adhesion, penetration and multiplication of a parasite in the target tissue of its host (Forattini 2001). Studies of virulence in different species of pathogenic fungi have contributed to the understanding of the pathogenesis of the resulting infections. The expression of extracellular enzymes, such as proteases, phospholipases, lipases and DNases, is associated with the virulence of strains that cause important mycoses, and the expression of these enzymes can provide clues about the nature of the relationship between the fungus and its host (Gácser et al. 2007).

The expression of extracellular enzymes has been increasingly studied in yeasts that are pathogenic to humans and other animals. Most of this work has been dedicated to the characterization of extracellular enzyme expression in the genus *Candida*, the most important yeast cause of nosocomial fungal infections. There are few published studies on the characteristics of enzymatic activity in *Trichosporon* spp. isolates (Ahearn et al. 1968; Teichert et al. 1989; Naglik et al. 2004). In the present study, exoenzymatic activity was detected in samples of *T. asahii* (n = 18) *T. inkin* (n = 10) *T. mucoides* (n = 4) and *T. ovoides* (n = 12). The extracellular enzymatic activity displayed by the samples of *Trichosporon* spp. varied according to the enzyme studied. Lipase activity was observed in 95.5% of the isolates studied, whereas phospholipase, protease and DNase activity was detected in 56.8%, 50% and 38.6% of the samples, respectively.

Lipases are responsible for the hydrolysis of triglycerides and the consequent production of glycerides and free fatty acids; phospholipases act on phospholipids present in cell membranes. The findings obtained in this study suggest that lipases, including phospholipases, are the major enzymes produced by *Trichosporon* spp. The literature indicates that lipase is an important factor in the pathogenicity of Basidiomycetes, specifically *Trichosporon* and *Malassezia* yeasts (Ran et al. 1993). The activity of lipases, including phospholipases, in these yeasts has been reported in strains isolated from bovine milk in nature (Dharmsthiti and Ammaranond 1997; Gonzáles et al. 2001; Melville et al. 2011). In another study, lipase activity was detected in all 48 isolates of *T. asahii* from patients hospitalized in Turkey (Dag and Cerikçioglu 2006).

Proteases are extracellular enzymes produced by pathogens, which play an important role in overcoming host immunological barriers. Some sophisticated virulence mechanisms act through the induction of apoptotic pathways (Forattini 2001). Groninger and Eklund ( 1996) pioneered work in the characterization of the proteases produced by *Trichosporon* yeasts (Groninger and Eklund 1996). In our study, 22 of the 44 tested *Trichosporon* isolates exhibited protease activity, and in 41% of the positive samples, this activity was strongly positive. However, a recent study reported that *T. asahii* isolates derived from both superficial and deep-seated infections did not express protease activity (Dag and Cerikçioglu 2006; Sun et al. 2012). These findings suggest that the protease expression profile of *Trichosporon* spp. is changing, reflecting the adaptation of pathogenic strains to their hosts. Deoxyribonucleases, also known as DNases, have received scant research attention in yeasts of the genus *Trichosporon*. Silvestre-Junior ( 2009) reported DNase activity in 84.3% of the *Trichosporon* isolates examined in a study conducted in Brazil. In the present study, only 38.6% of the samples were positive for DNase activity. The majority (58.8%) of the isolates that exhibited DNase activity were classified as strongly positive. Our results disagree with those of Silvestre-Junior ( 2009), who observed strongly positive DNase activity in only 10.2% of the samples (Silvestre-Junior 2009).

The induction of virulence mechanisms, such as the production of extracellular enzymes, is associated with factors related to the microorganism’s physiological stability. These factors include temperature, which may be relevant for the production and expression of enzymatic activity, considering the mesophilic nature of most known pathogens. In our research, the enzymatic activity of proteases and phospholipases was more efficient when the samples were incubated at 37°C compared with incubation at 25°C. Moreover, incubation at 37°C allowed the earlier detection of the expression of these enzymes (at 72 h). Our findings concerning proteases disagree with those reported by Melville et al. ( 2011), who did not observe significant differences between the protease activity of yeasts (including *Trichosporon* spp.) incubated at both temperatures. However, our phospholipase results agree with those of these authors, who also observed the largest number of samples with enzyme expression at 37°C (Melville et al. 2011).

In the present study, lipase activity was detected in a larger number of *Trichosporon* spp. isolates during incubation at room temperature. Loperena et al. ( 2012) reported higher lipase activity in 31 yeasts isolated from different environments of the Antarctic continent when they were incubated at 20°C rather than at 4°C. These findings suggest that the metabolic mechanisms of lipase production in yeasts are more independent of the substrate and the surrounding environment than previously assumed: both the mesophilic and the psychrophilic yeasts exhibited higher lipase expression when incubated at room temperature (Loperena et al. 2012).

Regardless of the enzyme in question, our results indicate that *Trichosporon* spp. have high growth potential at 37°C, which is a factor associated with the virulence of infectious agents. In isolation, this factor may not be responsible for the opportunistic infections caused by yeasts of this genus; however, in combination with other factors, such as the production of proteases and phospholipases, the capacity to grow at body temperature is compelling (Melville et al. 2011). Despite the high mortality rates associated with trichosporonosis, the relevance of extracellular enzymes to these cases cannot be determined without performing *in vivo* experiments to better clarify the role of these enzymes in disease pathogenesis. Moreover, fungal virulence factors are not the only decisive contributor to the degree of mortality observed in immunocompromised patients infected with *Trichosporon* yeasts; patient outcomes result from a combination of several factors (Giorgio 1995; Zuben 1997; Forattini 2001).

## Conclusions

Based on the results of this study, it can be concluded that there is a considerable predominance of lipases and phospholipases in the extracellular enzyme expression profiles of yeasts of the genus *Trichosporon*. The production of proteases and phospholipases is favored at 37°C, whereas room temperature favors the expression of lipases and DNases. Although the limitation of relating the data in the paper to in vivo pathogenesis, may be the temperature of the human body can stimulates the early secretion of proteases and phospholipases, which can be detected after three days of incubation. The detection of strongly positive samples suggests that many of the *Trichosporon* species that are considered pathogenic are not yet fully adapted to their hosts; however, these species will undergo an attenuation of virulence and a relationship of increasingly harmonic parasitism in the future.

## Methods

Forty-four samples of *Trichosporon* spp. were studied. The samples were classified in the collection as EPM001/2009 to EPM040/2012. Four strains were standard strains (*T. asahii* CBS 2479, *T. inkin* CBS 5585, *T. mucoides* CBS 7625, and *T. ovoides* CBS 7556), and the other 40 isolates were clinical samples maintained by periodic sampling in the Mycology Laboratory of the Departments of Cellular Biology and Microbiology, Immunology and Parasitology at the Federal University of São Paulo (UNIFESP). The isolates were previously identified by their phenotypic and genotypic profiles, as recommended by Rodriguez-Tudela et al. ( 2005). Cultures maintained in Sabouraud dextrose agar were used for all of the tests up to 72 h. To standardize the enzymatic activity readings, tests were performed at 1, 2, 3, 5, 7 and 15 days of incubation. The enzymatic activity (Pz) was determined by calculating the ratio of the diameter of the colony (dc) to the diameter of the halo produced (dcd). The results were classified as follows: negative (Pz = 1.0), positive (0.64 ≤ Pz < 1.0) or strongly positive (Pz < 0.64), as described by Price et al., 1982.

The enzyme activity assays followed the recommendations of Ruchel et al. ( 1982) for proteases, Price et al. ( 1982) for phospholipases, Muhsin et al. ( 1997) for lipases and López-Martínez et al. ( 1994) for DNases. The following substrates were used for the production of proteases, phospholipases, lipases and DNases: bovine serum albumin (fraction V, Sigma-Aldrich™, St. Louis, MO, USA), homogenized egg yolk, Tween 20 (20%) and DNase test agar (Oxoid™, Basingstoke, Hampishire, UK), respectively. A strain of *Candida albicans* (ICB-12A) was used as the positive control for the protease and phospholipase activity reactions. A strain of *Malassezia pachydermatis* (ICB-145) was the positive control for the lipase tests, and a strain of *Staphylococcus aureus* (ATCC 25923) was employed as a positive control for the DNase tests. Samples capable of producing a translucent halo (proteases), an opaque halo (phospholipases and lipases) or a halo of precipitate (DNases) were considered positive. All tests were performed in triplicate.

The statistical analyses were performed using the GraphPad Prism™ software, version 5.0. Student’s *t*-test and Fisher’s exact test were performed to compare the expression of enzyme activity at different times and temperatures. Significance was set at *p <* 0.05.

## Authors’ information

This paper is part of the HDLB’s thesis for the Postgraduate Program in Microbiology and Immunology of the Federal University of São Paulo (UNIFESP), São Paulo, SP, Brazil.
